# Our New Associates

**Published:** 1873-04

**Authors:** 


### OUR NEW ASSOCIATES.

THE additions to our editorial corps consist, as appears on the cover of this number, of Robert Battey, of Rome, as corresponding editor, and Drs. John G. Westmoreland, Ch. Rauschenberg and V. H. Taliaferro, of Atlanta, as associate editors.

Our readers are well acquainted with all of them as regular contributors of valuable papers to the pages of this journal. Their former labors are alike tokens of their valuable professional attainments, their ability as medical observers and writers, their studious, diligent and disinterested devotion to medical science and the highest interests of the profession. Praise of their respective individual merits on our part would be superfluous and out of good taste. The influence which their labors must necessarily exercise upon the intrinsic merit of the JOURNAL will very soon prove the value of their services as corresponding and associate editors.

Page(s) missing

